# Anti-Obesity Effects of *Pleurotus ferulae* Water Extract on 3T3-L1 Adipocytes and High-Fat-Diet-Induced Obese Mice

**DOI:** 10.3390/nu16234139

**Published:** 2024-11-29

**Authors:** Seulmin Hong, Seonkyeong Park, Jangho Lee, Soohyun Park, Jaeho Park, Yugeon Lee

**Affiliations:** 1 Food Functionality Research Division, Korea Food Research Institute (KFRI), Wanju-gun, Jeonbuk-do 55365, Republic of Korea; h.seulmin@kfri.re.kr (S.H.); p.seonkyeong@kfri.re.kr (S.P.); jhlee@kfri.re.kr (J.L.); shpark0204@kfri.re.kr (S.P.); jaehopark@kfri.re.kr (J.P.); 2 Department of Food Science and Technology, Chung-Ang University, Anseong, Gyeonggi-do 17546, Republic of Korea

**Keywords:** *Pleurotus ferulae*, high-fat diet, obesity, adipogenesis, polysaccharides

## Abstract

This study offers promising insights into the anti-obesity potential *of Pleurotus ferulae,* an edible mushroom valued in Asian cuisine for its nutritional benefits. A hot water extract of *P. ferulae* (PWE) administered to high-fat diet-induced obese mice over an 8-week period significantly reduced their body weight gain and fat accumulation. PWE not only improved the body weight metrics but also positively influenced the serum lipid profile of obese mice by lowering their total cholesterol and low-density lipoprotein cholesterol levels. In vitro studies using 3T3-L1 adipocytes showed that PWE inhibited adipocyte differentiation and lipid accumulation by downregulating key adipogenic transcription factors, particularly PPARγ and C/EBPα, as well as related lipogenic genes involved in fat synthesis and storage, such as Fabp4, Fasn, and Scd1. Chemical analysis revealed that PWE is rich in polysaccharides, which have been associated with various health benefits, including anti-obesity, anti-diabetic, and anti-cancer properties. These findings suggest that the bioactive compounds in PWE may serve as functional food components that could potentially be applied for the prevention and management of obesity and other metabolic disorders.

## 1. Introduction

Obesity is a prevalent metabolic disorder characterized by an increase in white adipose tissue mass, attributed to both adipocyte hypertrophy and hyperplasia [1]. Preadipocyte differentiation into mature adipocytes involves complex mechanisms regulated by key transcription factors, including peroxisome proliferator-activated receptor γ (PPARγ) and CCAAT/enhancer-binding protein (C/EBP) [2]. Both proteins play pivotal roles in the early stages of adipogenesis, with their cross-regulation being crucial for proper differentiation [3]. Specifically, PPARγ is considered indispensable for the formation of white adipocytes [4], considering its subsequent activation of genes related to free fatty acid uptake, intracellular transport, and lipid synthesis, including fatty acid-binding protein 4 (FABP4), fatty acid synthase (FASN), and ATP-citrate lyase (ACLY) [5]. Therefore, targeting lipogenic factors in adipose tissue and adipocytes could be an effective strategy for the prevention and management of obesity and its associated metabolic disorders.

Obesity is often associated with hyperlipidemia, a lipid disorder characterized by both quantitative and qualitative alterations in plasma lipoproteins [6]. The primary lipid abnormalities include elevated triglycerides, reduced high-density lipoprotein cholesterol levels, and elevated low-density lipoprotein cholesterol (LDL-C) levels [7]. Elevated serum triglycerides result from the increased production of hepatic very low-density lipoprotein particles and the impaired clearance of triglyceride-rich lipoproteins from the circulation [8]. Numerous studies have investigated the relationship between obesity and lipid profiles in animal models, focusing particularly on total cholesterol (TC) and LDL-C levels [9]. Notably, one study found that high-fat-diet (HFD)-induced obese mice showed significantly higher serum TC and LDL-C levels than mice on a normal diet [10,11]. Therefore, lipid profile alterations have been considered a critical factor in determining the anti-obesity effects of potential therapeutic options, including food components.

Dietary and medicinal mushrooms have been widely recognized for their extensive health benefits, which include immune system regulation and anti-viral, anti-diabetic, and anti-cancer effects [12]. Various species, such as *Lentinus*, *Auricularia*, *Hericium*, *Grifola*, *Flammulina*, *Tremella*, and *Pleurotus*, have demonstrated notable functional and nutritional properties [13]. Among these, *Pleurotus ferulae*, an edible mushroom prized for its culinary qualities, has been especially valued by consumers for its distinctive flavor and texture [14]. Beyond its culinary appeal, *P. ferulae* has shown promising medicinal properties, including anticancer activities [15]. However, research on its anti-obesity effects remains limited. The current study therefore sought to investigate the anti-obesity potential of *P. ferulae* using both an HFD-induced obese mouse model and a 3T3-L1 adipocyte cell line model.

## 2. Materials and Methods

### 2.1. Materials and Reagents

Dulbecco’s Modified Eagle Medium (DMEM, #LM001-05), fetal bovine serum (FBS, #S001-01), and bovine calf serum (BCS, #S003-01) were purchased from Welgene (Gyeongsan, Republic of Korea). Penicillin–streptomycin solution (10,000 U/mL) was obtained from Gibco (Carlsbad, CA, USA). Dimethyl sulfoxide (DMSO, #276855), 3-isobutyl-1-methylxanthine (IBMX, #I5879), dexamethasone (DEX, #D1756), insulin (#I6634), Oil Red O (ORO, #O1516) solution, gallic acid (#G7384), D-(+)-glucose (#G7021), and quercetin (#Q4951) were obtained from Sigma-Aldrich (St. Louis, MO, USA). Antibodies against PPARγ (16643-1-AP, Proteintech, Chicago, IL, USA), ATP-citrate lyase (ACLY, #15421-1-AP, Proteintech, Chicago, IL, USA), and α/β-tubulin (#2148, Cell Signaling Technology, Danvers, MA, USA) were used for Western blotting.

### 2.2. P. ferulae Extract Preparation

*P. ferulae* was cultivated in Chilgok-gun (Gyeongsangbuk-do, Republic of Korea) and harvested in February 2020. After harvesting, the mushrooms were washed, dried, and crushed. To prepare the *P. ferulae* water extract (PWE), the sample was mixed with distilled water at a 1:20 (*w*/*w*) ratio. Extraction was performed twice using a reflux cooling method at 50 °C for 3 h. The resulting extract was then filtered, freeze-dried and stored at 20 °C until further use. For the *P. ferulae* ethanol extract (PEE), the dried sample was extracted with 70% ethanol (100 g/L) twice using a reflux cooling method for 3 h at 50 °C. This extract was then filtered, concentrated under reduced pressure, freeze-dried, and stored at 20 °C until further use.

### 2.3. Animal Experiment

Five-week-old male C57BL/6 mice were purchased from Orient Bio Inc. (Seongnam, Gyeonggi-do, Republic of Korea) and housed under controlled conditions at 22 ± 2 °C with 50 ± 5% humidity and a 12 h light/dark cycle. This study adhered to ethical guidelines for animal experimentation and was approved by the Institutional Animal Care and Use Committee of the Korea Food Research Institute (Approval No: KFRI-M-23007; approved on 4 December 2023). After a 1-week acclimation period, the mice were divided into the following groups: a normal diet control group (ND), an HFD control group, and an HFD group supplemented with PWE. The HFD provided 60% of kcal from fat (D12492, Research Diets, Inc., New Brunswick, NJ, USA). PWE was dissolved in drinking water and administered orally at a dose of 100 mg/kg of body weight. Weight changes in the mice were monitored through weekly body weight measurements for 8 weeks. The mice’s body composition and fat mass were assessed using an InAlyzer (Medikors, Seoul, Republic of Korea). At the end of the feeding period, the mice were sacrificed for further analysis. Blood samples were collected, and serum was separated by centrifugation for lipid profiling analysis, conducted at OBEN Bio (Suwon, Republic of Korea) using a Beckman Coulter AU480 analyzer (Siemens, Germany).

### 2.4. Histological Analysis

Tissues were fixed with 3.7% paraformaldehyde (Biosesang, Yongin, Gyeonggi-do, Republic of Korea), embedded in paraffin wax, and sectioned sagittally to a thickness of 3 µm. Thereafter, the sections were stained with hematoxylin and eosin (H&E) to assess any morphological changes. Images of the stained adipose tissue were captured using a light microscope equipped with a camera (OBEN Bio). Image analysis was performed using ImageJ software (version 1.54d).

### 2.5. Cell Culture

Mouse 3T3-L1 preadipocytes were obtained from the American Type Culture Collection (Manassas, VA, USA) and maintained in high-glucose DMEM supplemented with 10% BCS and 1% penicillin–streptomycin solution at 37 °C in a 5% CO_2_ atmosphere. For differentiation, confluent cells were incubated in DMEM containing 10% FBS and a differentiation mix (MDI) consisting of 10 µg/mL insulin, 0.5 mM of IBMX, and 1 µM of DEX for 2 days. Subsequently, the medium was replaced every other day with insulin-supplemented DMEM (10 µg/mL). During differentiation, the cells were treated with either DMSO (control) or PWE for 8 days.

### 2.6. Cell Viability Assay

Cell viability was evaluated using the MTT assay. Briefly, the cells were seeded into a 96-well plate at a density of 1 × 104 cells per well. After 2 days, the differentiation of the cells was induced using MDI-supplemented FBS-DMEM and they were treated with various concentrations of PWE for 8 days. Following treatment, MTT stock solution (5 mg/mL) was added to each well followed by incubation for 2 h. After subsequently removing the medium, the resulting formazan crystals were dissolved in DMSO. The absorbance was then measured at 570 nm using a microplate reader (Molecular Devices, Sunnyvale, CA, USA).

### 2.7. ORO Staining

Lipid droplet accumulation was determined using ORO staining. Briefly, differentiated cells were washed with Dulbecco’s Phosphate-Buffered Saline and fixed with 3.7% paraformaldehyde (Biosesang) for 15 min. The cells were then washed with 60% isopropanol and subsequently stained with ORO solution for 20 min. Residual reagent was completely removed by washing the cells three times with distilled water, after which the stained cells were visualized using an Olympus IX73 light microscope (Olympus, Center Valley, PA, USA). To quantify lipid accumulation, the ORO stain was eluted using 100% isopropanol, and the absorbance was measured at 490 nm using a microplate reader (Molecular Devices).

### 2.8. Western Blot Analysis

Protein expression was confirmed using Western blotting. Briefly, the cells were lysed with RIPA buffer (#R0278, Cell Signaling Technology) containing phosphatase and protease inhibitor cocktails (Roche, Mannheim, Germany). Thereafter, the cell lysate was incubated on ice for 10 min and then centrifuged to collect the supernatant. The protein concentration was quantified using a BCA assay (iNtron Biotechnology, Inc., Seoul, Republic of Korea). Equal amounts of protein were mixed with 5× SDS-PAGE buffer (Biosesang), boiled at 95 °C for 5 min, and then cooled. Proteins were separated via SDS-PAGE and then transferred to polyvinylidene difluoride membranes (Bio-Rad, Hercules, CA, USA), which were then blocked with blocking buffer (#ML062-01, Welgene) for 1 h to prevent nonspecific binding. Subsequently, the membranes were incubated with primary antibodies at 4 °C for 12 h, followed by incubation with secondary antibodies at room temperature for 1 h. Immunoblots were developed using Pierce ECL Western Blotting Substrate (Thermo Fisher Scientific, Sunnyvale, CA, USA), and densitometry analysis was conducted using ImageJ to quantify protein expression.

### 2.9. RNA Analysis

RNA expression was evaluated using real-time polymerase chain reaction (PCR). Total RNA was extracted using the RNeasy Mini Kit (Qiagen, Hilden, Germany), and complementary DNA (cDNA) was synthesized using the cDNA Reverse Transcription Kit (#FSQ-301, TOYOBO Co., Ltd., Osaka, Japan). Real-time PCR was performed using FastStart Universal SYBR Green (Roche Molecular Biochemicals), specific primers, and equal amounts of each cDNA sample. The primer sequences used for RT-PCR are listed in Table 1. The thermal cycling conditions were as follows: initial denaturation at 95 °C for 10 min, followed by 40 cycles of 95 °C for 15 s, 60 °C for 1 min, and a final step at 65 °C for 5 s. The relative expression of each gene was normalized to the expression level of RPLP0 and calculated using the ∆∆Ct method.

### 2.10. Total Phenolic Content

The total phenolic content was determined using the Folin–Ciocalteu method [16]. Briefly, 0.1 mL of the sample or standard (gallic acid) was mixed with 0.1 mL of Folin–Ciocalteu phenol reagent (#F9592, Sigma-Aldrich) and incubated for 5 min at room temperature. Subsequently, 1 mL of 7% Na_2_CO_3_ solution and 0.4 mL of distilled water were added, and the mixture was incubated for 2 h at room temperature in the dark. The absorbance of the resulting solution was measured at 760 nm using a microplate reader (Molecular Devices). The results were expressed as gallic acid equivalents per 1 mg of dry weight (μg GAE/mg of dried weight).

### 2.11. Total Flavonoid Content

The total flavonoid content was determined using a colorimetric method with slight modifications [17]. Briefly, 0.1 mL of the sample or standard (quercetin) was mixed with 1 mL of diethylene glycol (H26456, Sigma-Aldrich) and 0.1 mL of 1 N NaOH. The mixture was allowed to react at 30 °C for 1 h. Subsequently, the absorbance of the reaction product was measured at 420 nm using a microplate reader (Molecular Devices). The flavonoid content of the sample was expressed in quercetin equivalent units (μg QE/mg of dried weight).

### 2.12. Total Polysaccharide Content

The total polysaccharide content was determined using the phenol–sulfuric acid method [18]. Briefly, a 0.1 mL aliquot of a 5% phenol solution (#P4557, Sigma-Aldrich) was added to 0.1 mL of the sample or standard (glucose) and thoroughly mixed. Thereafter, 0.5 mL of sulfuric acid (#258105, Sigma-Aldrich) was added to the mixture, which was incubated at room temperature for 10 min. The absorbance of the resulting product was measured at 490 nm using a microplate reader (Molecular Devices). The polysaccharide content was expressed as glucose equivalent units (μg GE/mg of dried weight).

### 2.13. Statistical Analysis

All experiments were conducted in triplicate, with the results being presented as mean ± standard deviation (mean ± SD). Statistical significance was assessed using a one-way analysis of variance followed by Tukey’s post hoc test for multiple comparisons or an unpaired two-tailed Student’s *t*-test using Prism 8 software (GraphPad Software, San Diego, CA, USA). A *p* value of 0.05 indicated statistical significance.

## 3. Results

### 3.1. Effects of the PWE on Body Weight Changes in HFD-Induced Obese Mice

**Figure 1:**
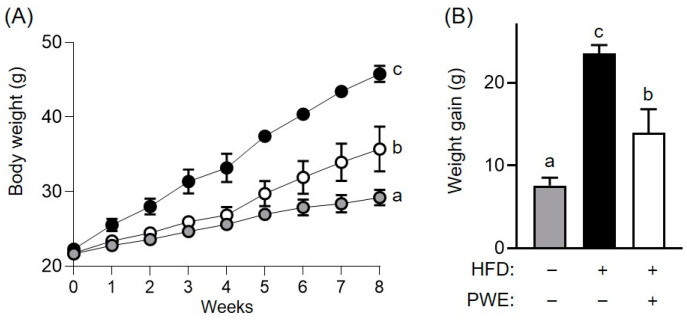
Anti-obesity effects of *Pleurotus ferulae* water extract (PWE) intake in high-fat-diet-induced obese mice. Alteration in body weight over 8 weeks (**A**) and weight gain after 8 weeks (**B**) in the high-fat-induced obese model with or without PWE intake. All results are expressed as mean ± standard deviation (SD) (*n* = 3). Different letters indicate significant differences (*p* < 0.05) determined via one-way analysis of variance followed by Tukey’s post hoc test.

ORO staining analysis was conducted to compare the inhibitory effects of PWE and PEE on lipid accumulation. Our results showed that PWE had a stronger potential to inhibit lipid accumulation than PEE (Supplementary Figure S1). Based on these findings, an animal study was conducted to evaluate the effects of *P. ferulae* on obesity using its water extract (PWE). Mice were fed a HFD supplemented with PWE for 8 weeks while the changes in their body weight were monitored. The HFD group exhibited significantly greater body weight gain than the ND group, whereas the PWE group showed significantly lower body weight gain than the HFD group (Figure 1A). Specifically, after 8 weeks on the HFD, the HFD group had an average weight gain of 23.5 g, whereas the PWE group showed a weight reduction of 14.0 g (Figure 1B).

### 3.2. Effects of PWE on Body Fat Mass and Adipocyte Area in HFD-Induced Obese Mice

**Figure 2:**
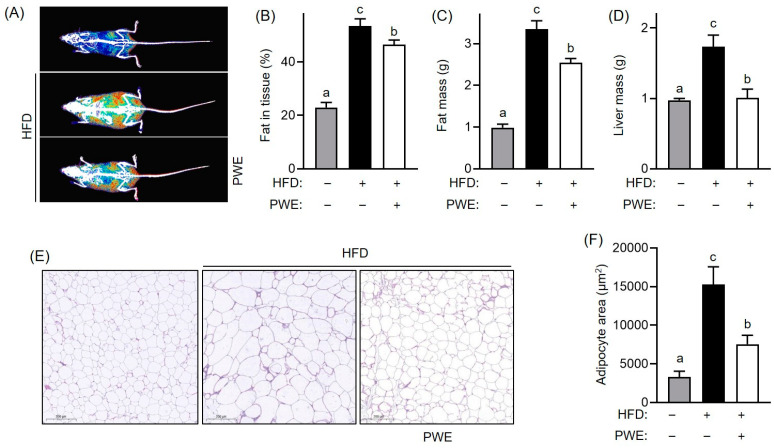
Effects of *Pleurotus ferulae* water extract (PWE) intake on tissue mass and morphological changes induced by a high fat diet. Alteration in body image (**A**), body fat content (**B**), fat mass (**C**), and liver mass (**D**). Hematoxylin and eosin (H&E) staining image (**E**) and adipocyte size (**F**) of epididymal white adipose tissue in a high-fat diet-induced obese model with or without PWE intake. All results are expressed as mean ± standard deviation (SD) (n = 3). Different letters indicate significant differences (*p* < 0.05) determined via one-way analysis of variance followed by Tukey’s post hoc test.

Consistent with the trend in body weight changes, the body fat mass measured using the InAlyzer was significantly greater in the HFD group (53.4%) than in the ND group (23%), with a lower increase observed in the PWE-supplemented group (46.5%) than in the HFD group (Figure 2A,B). Furthermore, PWE supplementation significantly reduced the tissue mass of adipose tissue (−23.9%) and liver (−41.8%) compared to the HFD group (Figure 2C,D). Additionally, H&E staining revealed that the adipocyte area in epididymal white adipose tissue was significantly larger in the HFD group (15,299.4 μm^2^) than in the ND group (3287.9 μm^2^). However, the PWE group (7560.9 μm^2^) showed a significant reduction in adipocyte size despite receiving a HFD (Figure 2E,F). These findings suggest that PWE effectively suppressed HFD-induced weight gain and adipocyte hypertrophy.

### 3.3. Effects of PWE on Serum TC and LDL-C Levels in HFD-Induced Obese Mice

**Figure 3:**
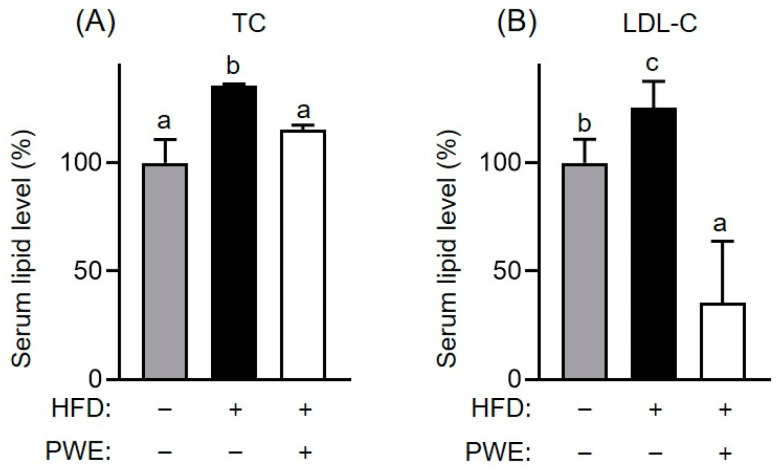
Effects of *Pleurotus ferulae* water extract (PWE) intake on lipid profiles following a high fat diet. Alterations in total serum cholesterol (**A**) and serum low-density lipoprotein cholesterol levels (**B**) in a high-fat-diet-induced obese model with or without PWE intake. All results are expressed as mean ± standard deviation (SD) (n = 3). Different letters indicate significant differences (*p* < 0.05) determined via one-way analysis of variance followed by Tukey’s post hoc test.

To further investigate the effects of PWE on lipid metabolism, the serum levels of TC and LDL-C were analyzed. Notably, both the TC and LDL-C levels were higher in the HFD group than in the ND group (Figure 3A,B). However, PWE treatment significantly reduced the TC and LDL-C levels in mice receiving the HFD. These results indicate that PWE intake effectively mitigated the lipid imbalances associated with HFD-induced obesity.

### 3.4. Effects of PWE on Lipid Accumulation in 3T3-L1 Adipocytes

**Figure 4:**
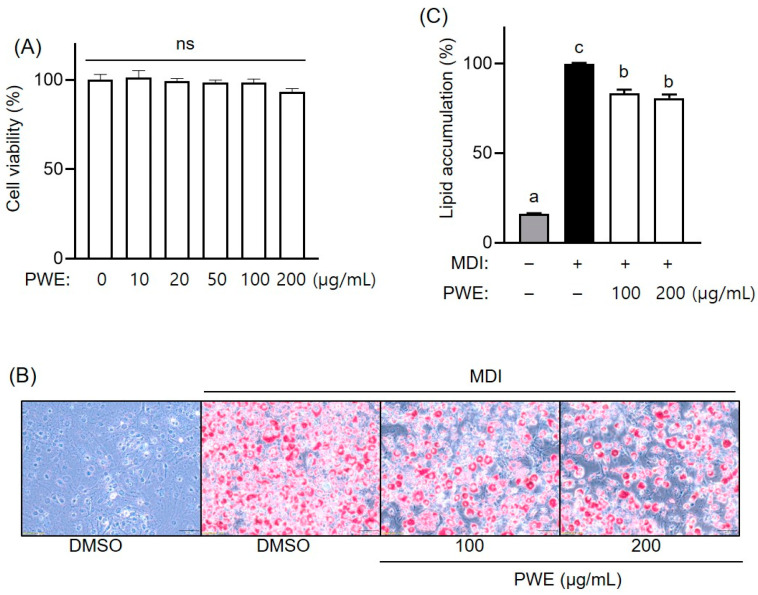
Effects of *Pleurotus ferulae* water extract (PWE) on cytotoxicity and lipid accumulation in 3T3-L1 cells. PWE cytotoxicity in 3T3-L1 adipocytes (**A**) was assessed using the MTT assay. Lipid droplet images were captured using microscopy (**B**), and lipid accumulation (**C**) after Oil Red O staining was measured in 3T3-L1 adipocytes with or without PWE treatment. All results are expressed as mean ± standard deviation (SD) (n = 3). Different letters indicate significant differences (*p* < 0.05) determined via one-way analysis of variance followed by Tukey’s post hoc test. ns, not significant.

The 3T3-L1 preadipocyte differentiation model was utilized to investigate the effects of PWE on obesity at the cellular level. First, we conducted a cell viability assay using the MTT reagent. After the supplementation of PWE at various concentrations (0–200 μg/mL) during differentiation, no cytotoxicity was observed in the PWE-treated groups (Figure 4A). Based on these results, treatment concentrations of 100 and 200 μg/mL were selected for further experiments. Subsequently, the inhibition of lipid accumulation was assessed using ORO staining (Figure 4B). More lipid droplets were observed in the differentiated group than in the control group. However, PWE treatment dose-dependently decreased lipid accumulation, with the group treated with 200 μg/mL of PWE showing an approximately 20% lower lipid accumulation than the DMSO-treated group (Figure 4C). These findings indicate that PWE effectively inhibited lipid accumulation during adipocyte differentiation without affecting cell viability.

### 3.5. Effects of PWE on Lipid Metabolism-Related Factors in 3T3-L1 Adipocytes

**Figure 5:**
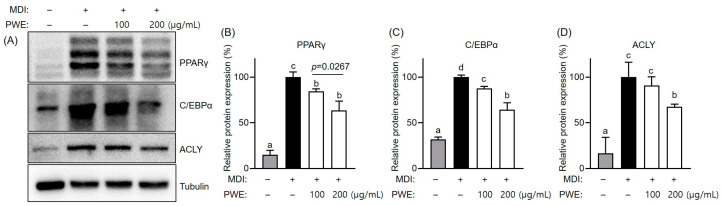
Effects of *Pleurotus ferulae* water extract (PWE) on adipogenesis-associated protein expression in 3T3-L1 adipocytes. Band images (**A**) and quantification of PPARγ (**B**), C/EBPα (**C**) and ACLY (**D**) by Western blot assay in 3T3-L1 adipocytes with or without PWE treatment. All results are expressed as mean ± standard deviation (SD) (n = 3). Different letters indicate significant differences (*p* < 0.05) determined via one-way analysis of variance followed by Tukey’s post hoc test. Statistical significance (*p* value) was determined using the unpaired two-tailed Student’s *t*-test.

**Figure 6:**
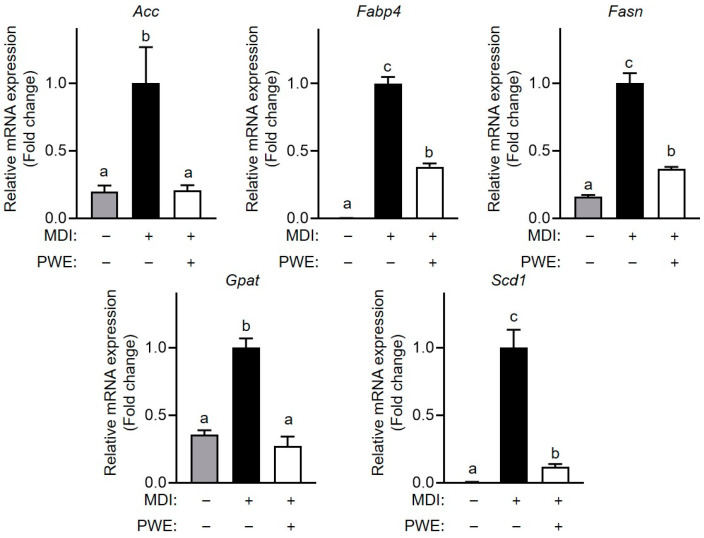
Effects of *Pleurotus ferulae* water extract (PWE) on adipogenesis-associated gene expression in 3T3-L1 adipocytes. qPCR analysis was conducted to quantify the mRNA expression of Acc, Fabp4, Fasn, Gpat, and Scd1 in 3T3-L1 adipocytes with or without PWE treatment. All results are expressed as mean ± standard deviation (SD) (n = 3). Different letters indicate significant differences (*p* < 0.05) determined via one-way analysis of variance followed by Tukey’s post hoc test.

To further assess the effects of PWE on lipid accumulation, the expression of the key proteins involved in adipogenesis and lipogenesis was analyzed. Our results showed that PPARγ, C/EBPα and ACLY expression significantly increased during adipocyte differentiation. However, differentiated adipocytes treated with PWE significantly reduced the expression levels of these proteins (Figure 5A,B). Additionally, the mRNA expression levels of genes essential for adipogenesis and lipogenesis, such as Acc, Fabp4, Fasn, Gpat, and Scd1, were upregulated during differentiation. However, PWE treatment decreased the expression levels of such genes (Figure 6). Collectively, these findings suggest that PWE effectively reduced lipid accumulation in adipocytes by suppressing the expression of genes associated with lipid metabolism during differentiation.

### 3.6. Analysis of the Major Bioactive Compounds in PWE

Finally, the total phenolics, flavonoids, and polysaccharides in PWE were quantified using colorimetric methods to identify the bioactive components in *P. ferulae*. Notably, phenolics and flavonoids were undetectable in the PWE; however, PWE contained a significant number of polysaccharides (662.2 μg GE/mg of dried weight; Table 2). In contrast, the PEE also lacked phenolics and flavonoids but contained lower polysaccharide levels than the PWE. This finding suggests that the high polysaccharide content in PWE may play a significant role in its potential effects on lipid accumulation and that polysaccharides are likely a key component in the health benefits of *P. ferulae*.

## 4. Discussion

Obesity is characterized by an increase in body weight due to excessive fat accumulation [19]. This condition not only affects physical appearance but also significantly contributes to the development of metabolic diseases, such as diabetes and cardiovascular diseases [20]. These metabolic disturbances have been closely associated with insulin resistance and chronic inflammation, which together disrupt lipid homeostasis [21]. Consequently, this vicious cycle has been considered a major risk factor for numerous health issues, underscoring the need for strategies that effectively address and manage excessive fat accumulation and its systemic effects.

Adipose tissue, an insulin-sensitive peripheral organ, plays a critical role in maintaining energy homeostasis by secreting various hormones [22]. Adipogenesis involves a complex transcriptional cascade wherein transcription factors, such as PPARγ, are sequentially activated at the molecular level [23]. Additionally, PPARγ works synergistically with C/EBPα to support the synthesis and transport of free fatty acids (FFAs), processes that are vital for lipid metabolism and the proper functioning of adipose tissue in energy regulation [24]. However, under conditions of obesity, these transcription factors, PPARγ and C/EBPα, not only enhance insulin-mediated glucose uptake but also contribute to increased FFA production. Therefore, targeting these transcriptional pathways in differentiating adipocytes represents a promising therapeutic strategy for addressing obesity [25]. Our findings revealed that PWE treatment significantly reduced the protein expression levels of PPARγ and C/EBPα in differentiated 3T3-L1 cells, a widely used model for studying adipocyte differentiation. Additionally, PWE suppressed the insulin-induced activation of lipogenic genes, lipid transport, and lipid droplet formation. These findings suggest that PWE exerts anti-adipogenic and anti-lipogenic effects by modulating the key transcription factors involved in insulin-stimulated adipocyte differentiation. Furthermore, an 8-week administration of PWE in high-fat-diet-induced obese mice resulted in significant reductions in body weight, fat mass, and serum levels of TC and LDL-C.

Recent studies have highlighted the anti-obesity potential of various edible mushrooms, attributing their effects to bioactive compounds [26,27]. For example, the phenolic-rich fractions of *Melanoleuca stridula*, *Chlorophyllum agaricoides*, and *Tricholoma populinum* showed higher radical-scavenging activity than their organic solvent extracts [28,29]. Moreover, a phenolic-rich extract of *Pleurotus eryngii* was reported to exert anti-inflammatory activity by inhibiting NF-κB signaling-mediated inflammation pathways [30]. Fiber intake from *Agaricus bisporus* mushrooms significantly reduced serum cholesterol and LDL levels by upregulating LDL receptor expression [31]. The administration of Yamabushitake mushroom (*Hericium erinaceus*) in HFD-induced obese mice significantly decreased their body weight gain and fat weight by modulating the expression of lipid metabolic genes, including PPARα [32]. Another study found that the consumption of a HFD supplemented with alkaloid mushrooms from *Lentinus edodes* reduced HFD-induced increases in serum triglyceride and LDL levels [33]. Additionally, *P. ostreatus* has been shown to suppress lipid synthesis by modulating the gut microbiota, thereby improving lipid and glucose metabolism in HFD-supplemented mouse models [34]. Overall, these findings suggest that supplementation with *P. ferulae,* which is rich in polysaccharides, could be a promising strategy for alleviating obesity and its associated metabolic dysregulation. However, further research is needed to fully understand the specific molecular pathways through which *P. ferulae* reduces lipid accumulation in mature adipocytes and in vivo models.

Although phenolic acids and flavonoids have been considered the most common bioactive components in mushroom species, other functional compounds, including sterols, terpenoids, and polysaccharides, have also been found in mushrooms [35,36]. Polysaccharides, in particular, have garnered significant attention for their anticancer and immunomodulatory activities [37]. Furthermore, polysaccharides have been shown to alter gut microbiota composition, support the growth of beneficial bacteria, and reduce fat accumulation in obese mice, thereby promoting weight reduction [38]. Evidence also shows that they are capable of reducing oxidative stress and improving lipid profiles in diabetic mice by lowering triglyceride and TC levels [39]. Furthermore, recent clinical trials have suggested that polysaccharides can act as prebiotics, positively influencing the composition of the human gut microbiota [40]. Several polysaccharides with lipid-lowering effects, including glucans, heteroglycans, fucoidan, ulvan A, ulvan B, gentiobiose, and agaropectin, have been identified [41]. In addition, pectic-type polysaccharides such as rhamnogalacturonan-I (RG-I) and homogalacturonan (HG) have demonstrated potential in addressing metabolic disorders, including insulin resistance, inflammation, and diet-induced obesity [42]. Interestingly, the lipid-lowering activity of polysaccharides appears to be closely linked to their monosaccharide composition. For example, (1→3,1→4)-β-D-glucan and arabinoxylan can inhibit the reabsorption of bile acids in the large intestine, promoting bile salt excretion [43]. This process stimulates the use of plasma cholesterol for bile synthesis, effectively reducing blood TC and LDL-C levels. Additionally, polysaccharides with higher molecular weights tend to form more viscous gels, which increase the viscosity within the gastrointestinal tract [44]. This property restricts the diffusion of lipids and lipases, thereby slowing the lipolytic process and reducing blood lipid levels. Accordingly, exploring the polysaccharide components in PWE with lipid-lowering effects could provide valuable insights and significant therapeutic opportunities for managing metabolic disorders.

Consequently, polysaccharides from natural sources have been attracting considerable attention owing to their potential to improve various metabolic disorders, including obesity. In the current study, we confirmed that PWE was rich in polysaccharides, suggesting that its anti-obesity effects may be attributed to these compounds. However, further analysis is necessary to identify the individual active components and clarify their mechanisms of action. Conducting liquid chromatography–mass spectrometry analysis to isolate and characterize the polysaccharides present in PWE is essential. Such efforts could spearhead the application of mushroom polysaccharides in the functional food industry and support the development of targeted functional ingredients for obesity prevention and management.

## 5. Conclusions

Our findings suggest that *P. ferulae* effectively alleviated HFD-induced obesity and its associated metabolic disturbances. Using both animal and cellular models, we demonstrated that PWE supplementation significantly reduced body weight, fat mass, and the serum levels of TC and LDL-C in HFD-induced obese mice. Furthermore, PWE supplementation decreased lipid accumulation and adipocyte hypertrophy in white adipose tissue. In 3T3-L1 adipocytes, PWE inhibited lipid accumulation, reduced lipid droplet formation, and suppressed the expression of key adipogenic and lipogenic transcription factors, including PPARγ and C/EBPα. A further compound analysis of PWE revealed high levels of polysaccharides, which have been previously shown to exhibit anti-obesity, antioxidant, anti-inflammatory, and metabolic regulatory properties. Given the observed effects in our study, polysaccharides appear to be promising active components that account for the beneficial impact of PWE on lipid metabolism and adipocyte regulation. The influence of PWE on both systemic lipid regulation and cellular lipid metabolism presents a promising pathway for developing functional food ingredients targeting obesity and metabolic health.

## Figures and Tables

**Table 1:** Primer sequences used during RT-PCR analysis.

Origin	Gene	Direction	Sequence (5′-3′)
Mouse	Acc	forward	TCTATTCGGGGTGACTTTC
		reverse	CTATCAGTCTGTCCAGCCC
	Fabp4	forward	GGGAACCTGGAAGCTTGTCT
		reverse	ACTCTCTGACCGGATGGTGA
	Fasn	forward	AGAAGCCATGTGGGGAAGATT
		forward	AGCAGGGACAGGACAAGACAA
	Gpat	forward	GTAGTTGAACTCCTCCGACA
		forward	ATCCACTACCACTGAGAGGA
	Scd1	forward	TTCTTGCGATACACTCTGGTGC
		reverse	CGGGATTGAATGTTCTTGTCGT
	Rplpo	forward	GTGCTGATGGGCAAGAAC
		reverse	AGGTCCTCCTTGGTGAAC

**Table 2:** Total phenolic acid content, total flavonoid content, and total polysaccharide content of *Pleurotus ferulae* water extract (PWE) or *Pleurotus ferulae* ethanol extract (PEE).

	PWE	PEE
Total phenolic content (μg GAE)/mg dried weight)	Not detected	Not detected
Total flavonoid content (μg QE)/mg dried weight)	Not detected	Not detected
Total polysaccharide content (μg GE)/mg dried weight)	662.17 ± 8.78	549.59 ± 3.89

GAE; gallic acid equivalents, QE; quercetin equivalents, GE; glucose equivalents.

## Data Availability

The data presented in this study are available upon request from the corresponding author.

## Supplementary Materials

The following supporting information can be downloaded at: https://www.mdpi.com/article/10.3390/nu16234139/s1, Figure S1: Effects of *Pleurotus ferulae* water extract (PWE) or Pleurotus ferulae ethanol extract (PEE) on lipid accumulation in 3T3-L1 cells.
