# Supplementary material for: Anti-Obesity Effects of Pleurotus ferulae Water Extract on 3T3-L1 Adipocytes and High-Fat-Diet-Induced Obese Mice
Source: Nutrients. 2024 Nov 29;16(23):4139. doi: 10.3390/nu16234139 (PMC11644537; doi:10.3390/nu16234139)
Supplement: Supplementary file 1 [file nutrients-16-04139-s001.zip › nutrients-3338618-supplementary.docx]

**Supplementary data**


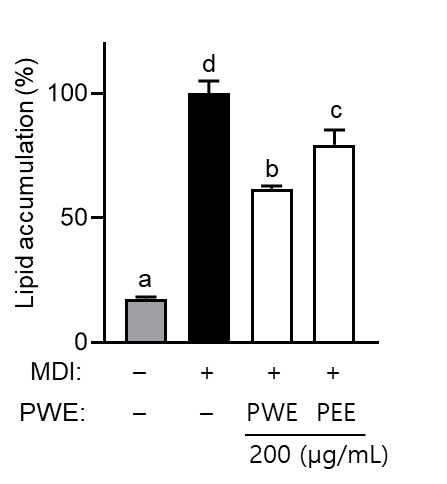


**Supplementary Figure S1. Effects of *Pleurotus ferulae water extract* (PWE) or *Pleurotus ferulae ethanol extract* (PEE) on lipid accumulation in 3T3-L1 cells.**

Lipid accumulation was measured in 3T3-L1 adipocytes following Oil Red O staining, both with and without PWE or PEE treatment. Results are expressed as the mean ± standard deviation (SD) (n = 3). Different letters indicate significant differences (p < 0.05), as determined by one-way analysis of variance (ANOVA) followed by Tukey’s post hoc test.
